# Homogeneous shear distribution improves NK-92 cell cytotoxicity in a clinically relevant 2 L membrane-stirred bioreactor

**DOI:** 10.3389/fbioe.2026.1804945

**Published:** 2026-03-23

**Authors:** Valentin von Werz, Yasemin van Heuvel, Matthias Hadrbolec, Michael Wagner, Philipp Eibl, Florian Huber, Moritz Meyer, Patrick Bongartz, Christian Witz, Oliver Spadiut

**Affiliations:** 1 Research Division Integrated Bioprocess Development, Institute of Chemical, Environmental and Bioscience Engineering, Technische Universität, Wien, Austria; 2 BioThrust GmbH, Aachen, Germany; 3 SimVantage GmbH, Graz, Austria

**Keywords:** bioreactor, computational fluid dynamics, fed-batch, membrane stirrer, NK-92, pitched-blade, shear force, cytotoxicity

## Abstract

The immortalized NK-92 cell line is widely used to study natural killer (NK) cell biology and develop immunotherapies. NK-92 cells exhibit strong cytotoxic activity against tumor and virus-infected cells and are often used as a functional surrogate for primary NK cells. Beyond research applications, NK-92 cells are currently being evaluated in clinical trials as an allogeneic, off-the-shelf cell therapy. NK cells are typically cultured in static systems, which limits scalability. For clinical and commercial applications, large-scale cell expansion requires scalable platforms, such as bioreactors. However, conventional bubble-aerated bioreactors generate shear stress and foam, which can impair proliferation during prolonged culture and compromise cell quality. To address these limitations, a membrane-based stirring and aeration system was compared to a conventional pitched-blade impeller with microsparger aeration in 2 L stirred-tank bioreactors. NK-92 cells were expanded from static pre-cultures into shake flasks and subsequently inoculated into each bioreactor with continuous feeding to maintain ideal nutrient supply. Both systems supported comparable growth, viability, and metabolic profiles. Cells showed comparable growth, viability and metabolism profile in both systems. However, cells expanded in the membrane-based system exhibited markedly higher cytotoxicity and cytotoxic capacity. Computational fluid dynamic simulations of both systems suggest that this observation is likely attributable to the more homogeneous shear distribution in the membrane-stirred setup compared to the pitched-blade configuration. Overall, this work presents a novel cultivation method to produce highly cytotoxic NK-92 cells in a well scalable stirred-tank bioreactor platform for allogeneic off-the-shelf cell therapy.

## Introduction

Manufacturing of cell and gene therapeutics is still dominated by two-dimensional or wave-based expansion platforms, such as static flasks, rocking bags, stacked cell factories, or G-Rex systems which are labor-intensive, require frequent manual handling, and limit scalability (Dias et al., 2024). Although these systems are supported by well-established good laboratory practices and suitable for good manufacturing practice (GMP) facilities (Wang et al., 2023; El-Kadiry et al., 2021), the rapid growth of cellular immunotherapies, particularly in immune oncology, is creating an urgent need for more scalable and economically viable production strategies (Cellular Immunotherapy Market, 2025; Cellular Immunotherapy Market Size, 2025; CAR T, 2025). The shift toward allogeneic therapies further amplifies this demand, as clinical manufacturing will require robust, large-scale expansion technologies such as stirred-tank bioreactors (Zeh et al., 2024).

Most currently approved immune cell therapies rely on autologous chimeric antigen receptor (CAR) T cells. Seven such products have been authorized by the U.S. Food and Drug Administration (FDA), and more than 1,700 clinical trials are ongoing worldwide (Search ClinicalTrials, 2025a). However, CAR-T therapies remain constrained by high production costs, limited scalability, and the risk of life-threatening graft-versus-host disease (GVHD), restricting their broad applicability (Depil et al., 2020). Natural killer (NK) cells have emerged as a promising alternative, offering a more favorable safety profile, intrinsic allogeneic compatibility, and the ability to target both hematologic malignancies and solid tumors (Wu et al., 2025). Although CAR-NK development began approximately a decade later than CAR-T, with the first reports published in 2009 (Altvate et al., 2009; Boissel et al., 2009), clinical evidence has since demonstrated their potential, including a landmark study in 2020 showing efficacy against CD19-positive lymphoid tumors (ClinicalTrials.gov: NCT03056339) (Liu et al., 2020). While no NK cell therapy has yet received FDA approval, 176 CAR-NK clinical trials with primary NK cells and the NK-92 cell line are registered, and this number is expected to grow substantially in the coming years (Search ClinicalTrials, 2025b).

The increasing demand for both T- and NK-cell therapies underscores the need for higher production yields and reduced manufacturing costs providing solutions to cost of goods challenges. Scalable three-dimensional allogeneic culture systems that support multi-dose production from a single batch will be essential. For example, generating approximately ten therapeutic doses from a single 10-L bioreactor run could reduce per-dose manufacturing costs by one order of magnitude. Further intensification toward cell densities of ∼1 × 10^7^ cells/mL could, in theory, reduce manufacturing costs by up to two orders of magnitude compared with current processes, where the cost per dose (×110^6^–1 × 10^9^ cells) remains in the range of $120,000–180,000 (Rady et al., 2025; Chen et al., 2025; Jenkins and Farid, 2018; Hartmann et al., 2017).

Conventional microsparged stirred-tank bioreactors pose significant challenges for large-scale expansion of immune and stem cells. At larger volumes, bubble bursting, turbulence, and foam formation introduce shear forces that can damage these highly shear-sensitive cells, which are considerably more delicate than production lines such as Chinese hamster ovary (CHO) (Ma et al., 2004; Hu et al., 2008; Flynn et al., 2024). While shear- or foam-reducing additives such as poloxamers or silicone-based antifoams can mitigate these effects, their use may negatively affect product quality, reduce yield, and introduce additional process risks (Flynn et al., 2024; Velugula-Yellela et al., 2018). Membrane-based gas-transfer systems offer a promising alternative by enabling efficient, bubble-free aeration and eliminating the need for antifoam or surfactants, thereby offering a better environment for the expansion of sensitive cell types (Contreras et al., 1999; Frahm et al., 2009; Bongartz et al., 2023). Their effectiveness is already demonstrated in membrane-based static culture platforms such as the G-Rex, which has become a gold standard for GMP-compliant immune cell manufacturing (Wang et al., 2024). Motivated by these considerations, we performed a head-to-head comparison of two bioreactor configurations for NK-92 expansion in 2 L stirred-tank systems in fed-batch mode: a conventional setup with pitched-blade impellers and a microsparger, and a novel system integrating membrane-based aeration and agitation.

## Materials and methods

### Static cultivation

NK-92 cells (ACC 488, Leibniz Institute DSMZ-German Collection of Microorganisms and Cell Cultures GmbH, Braunschweig, Germany) were cultivated at standard cultivation conditions (37 °C and 5% CO_2_ in a humidified incubator), in the cultivation medium, minimal essential medium alpha modification (αMEM; Gibco, Paisley, United Kingdom) supplemented to a final concentration of 1.15 mM L-Arginine (Carl Roth, Karlsruhe, Germany), 2.31 mM L-Glutamine (Carl Roth, Karlsruhe, Germany), 0.286 mM L-Serine (Thermo Fisher Scientific, Waltham, MA, United States of America), 0.194 mM i-inositol (Sigma-Aldrich, St. Louis, MO, United States of America), 23.2 mM D-Glucose (Carl Roth, Karlsruhe, Germany), 24 μM beta-mercaptoethanol (Sigma-Aldrich, St. Louis, MO, United States of America), Insulin-Transferrin-Selenium (Gibco, Grand Island, NY, United States of America) 17.21 μM, 0.69 μM and 0.39 μM, respectively, 2.2 g/L Sodium bicarbonate (Sigma-Aldrich, St. Louis, MO, United States of America), 500 IU/mL recombinant premium grade interleukin-2 (IL-2; Miltenyi Biotech, Bergisch Gladbach, Germany) and 10% type AB, off-the-clot human serum (Pan Biotech, Aidenbach, Germany). A batch cultivation was seeded in T flasks (Starlab, Brussels, Belgium) at a concentration of 2.5 × 10^5^ cells/mL and was supplemented with IL-2 every second day without media exchange or addition. Cells were seeded in T-25 flasks after thawing and then progressively expanded to T-175 flask size.

To assess the cytotoxicity of the NK-92 cells, K-562-GFP (K562) cells (CCL-243-GFP, American Type Culture Collection, Manassas, VA, United States of America) were maintained at standard cultivation conditions in a Roswell Park Memorial Institute 1,640 medium (RPMI; Gibco, Grand Island, NY, United States of America) containing 5% heat-inactivated fetal bovine serum (FBS, Gibco, Thermo Fisher Scientific, Waltham, MA, United States of America). Regular subculture of K562 cells was done at a seeding concentration of 1 × 10^6^ cells/mL in T75 flasks. All cells were cultured in an antibiotic-free culture and routinely confirmed to be *mycoplasma* negative using the Mycostrip *Mycoplasma* Detection Kit (Invivogen, Toulouse, France).

### Benchtop bioreactor cultivation

**FIGURE 1:**
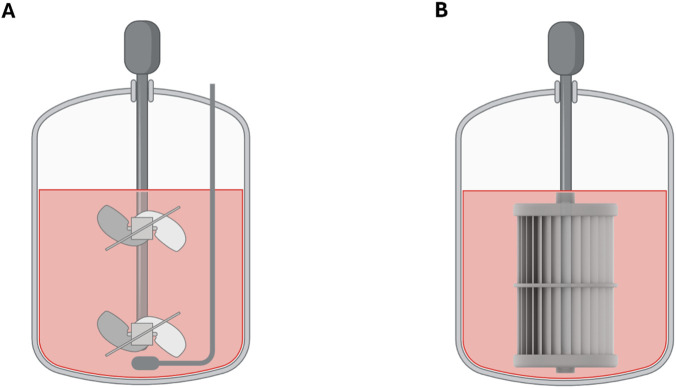
Schematic representation of the 2 L bioreactor assemblies with **(A)** pitched-blade impeller with microsparger and **(B)** the membrane stirrer. Figure was created with biorender.com.

Bioreactor cultivations were conducted in 2 L working volume UniVessel glass vessels (Sartorius, Göttingen, Germany) controlled by Labfors 5 (Infors HT, Bottmingen, Switzerland) controllers. Each vessel was equipped with either two, 30° 3-blade pitch-blade impellers and a microsparger for aeration or the membrane-based aeration and impeller (membrane stirrer) and fluid wave coupling combination (Comfy*Cell*, BioThrust GmbH, Aachen, Germany; Figure 1; Supplementary Figure S1; Supplementary Table S1). The pH and optical dissolved oxygen (dO) were analyzed using inline sensors (both Hamilton, Bonaduz, Switzerland). The cultures were heated with external heating blankets, and the temperature monitoring was performed using the built-in Pt100 probe. The inoculum was expanded in 250 mL shake flasks (Corning, Corning, United States of America) on an orbital shaker (orbit diameter 20 mm) at 120 rpm under standard culturing conditions following static expansion. The cultivation medium in the pre-culture flasks was replenished one or 2 days prior to inoculation for membrane stirrer run or for the pitched-blade run, respectively. The culture in the bioreactor was seeded at 2.5 × 10^5^ NK-92 cells/mL in 600 mL starting volume (80 rpm using the pitched blade impeller and 40 rpm using the membrane stirrer element, both at a kLa of 0.3 h^-1^, 37 °C) with IL-2 supplementation every second day. A total gas flow rate of 12 mL/min (0.02 vessel volumes per minute) was achieved by a constant pressurized air flow of 2 mL/min, varying pure oxygen flow to maintain a dO level of 60%, varying pure CO_2_ flow to sustain 5% CO_2_ level in the off-gas and varying pure nitrogen flow to reach the total gas flow rate, all controlled by mass flow controllers. The off-gas left the reactor through a chilled off-gas cooler and its O_2_, CO_2_ and humidity content were analyzed by a BlueInOne Cell gas analyzer (BlueSens, Herten, Germany). The pH was maintained at 7.35 (±0.05) with the addition of fresh cultivation medium delivered through the integrated pump system of the controller. In none of the experiments, antifoam or any shear-protecting agents was added to the cultures. To the initial 600 mL batch culture, a total of 1,000 mL fresh medium was added to the bioreactor during the fed-batch phase. The culture was maintained at 1,600 mL working volume after all media feed was added to the culture, until no further growth was observed.

### Cell counting and viability

Cell count and viability were measured by flow cytometry on a CytoFLEX S (Beckman Coulter, Brea, United States of America). 150 μL of cell suspension was mixed with 50 µL of cell counting beads (Invitrogen, Thermo Fisher Scientific, Waltham, United States of America) and 50 µL of 7 μg/mL 7-AAD (Invitrogen, Thermo Fisher Scientific, Waltham, United States of America) fluorescent stain for live-dead cell discrimination. The viable cell concentration was calculated as described in Equation 1.

$$Viable\;cell\;concentration\;\left[\frac{cells}{µL}\right]=\frac{\left({7AAD}^{-}\text{event}\;\text{count}\;x\;counting\;bead\;volume\right)}{\left(counting\;bead\;count\;x\;cell\;volume\right)}\;x\;counting\;bead\;concentration$$ (1)

Equation 1 Formula for the calculation of the viable cell concentraiton as instructed by the manufacturer of the counting beads. Cell count and bead count are determined by the respective event count in the gates by flow cytometry.

Size calibration beads for flow cytometry (Invitrogen, Thermo Fisher Scientific, Waltham, United States of America) were used to perform a size calibration that enabled the gating of particles based on the beads’ reference size. This calibration step minimized background signals in live-dead cell discrimination, which primarily resulted from protein aggregation in the medium, by excluding particles smaller than 8 μm, approximately half the size of an NK-92 cell which is 10–12 µm (Klingemann, 2023). Afterwards, viable cells were gated with the side-scatter signal, including only 7-AAD^-^ events. The bead count was gated according to the manufacturer´s protocol in a fluorescent channel (660 ± 10 nm detector) unaffected by other stains used in the analysis.

### Cytotoxicity and cytotoxic capacity determination

Cytotoxicity of NK-92 cells was assessed by co-culturing with K562 cells for 4 h, at an E:T ratio of 1:1. Both cell types were seeded in triplicates at a concentration of 1 × 10^5^ cells/well in 96-well V-bottom plates (Thermo Fisher Scientific, Waltham, United States of America) in 200 µL αMEM medium without serum and IL-2. Additionally, one negative control of both cell types containing the same cell amount (2 × 10^5^ cells/well) was seeded to assess spontaneous death. After 4 h, the cells were collected by centrifugation (300 × *g*, 5 min), washed in a FACS buffer (containing PBS +1% bovine serum albumin (Carl Roth, Karlsruhe, Germany) + 50 µM NaN_3_ (Thermo Fisher Scientific, Waltham, United States of America)), collected again (300 × *g*, 5 min), then stained with Annexin-V (BD Biosciences, Franklin Lakes, United States of America) for apoptosis. The reaction was carried out by adding 100 µL annexin binding buffer (ABB) (Invitrogen, Thermo Fisher Scientific, Waltham, United States of America) to each well, (which contained 0.625 µL Annexin-V) and incubated for 15 min at room temperature in the dark. Afterwards 150 µL ABB was added and cells were collected by centrifugation. After removal of the supernatant cells were resuspended in 250 µL 7-AAD in ABB to a final concentration of 1 μg/mL for the second staining step, where Annexin^+^ 7-AAD^-^ events indicated early apoptotic cells, Annexin^+^ 7-AAD^+^ events indicated late apoptotic cells, and Annexin^−^ 7-AAD^+^ events indicated necrotic cells. After the second staining, the cells were immediately analyzed by flow cytometry. For each measurement, a minimum of 10,000 events were recorded in the gate of live K562 or NK-92 cells, respectively.

Cytotoxicity was calculated by quantifying the loss of viable target cells in the co-culture with NK-92 cells, compared to the negative controls as recommended by the distributor of the target cells (Equation 2) (Dias et al., 2024; McWilliams-Koeppen and Clinton, 2022). Therefore, the count of early apoptotic, late apoptotic, and necrotic K562 cells were used from the co-culture sample and the negative control sample.

$$Cytotoxicity\;\left[\%\right]=\frac{\left(100\%-live\;K562\left[\%\right]\right)-\left(100\%-live\;{K562}_{control}\;\left[\%\right]\;\right)}{live\;{K562}_{control}\;\left[\%\right]}\;x\;100$$ (2)

Equation 2 Formula for the determination of absolute cytotoxicity from flow cytometry measurements where “live K562” are 7-AAD and Annexin-V double-negative effector events in co-culture wells, and “live K562_control_” are the 7-AAD and Annexin-V double-negative effector cells in the control wells.

In addition, the cytotoxic capacity was calculated to describe the potential of the whole culture to kill a target cell within 4 h of co-culture (Equation 3).

Cytotoxic capacity cells=cytotoxicity %x total viable cells number of cells (3)

Equation 3 Formula for the calculation of the cytotoxic capacity of the culture, where the cytotoxicity is multiplied by the total viable cell count of the culture. The result is a number of cells that have the capacity to eliminate one target cell.

To compare the overall productivity between media formulations and process modes, space-time yield was calculated as reported previously (Wang et al., 2023) (Equation 4).

$$\text{Space}-\text{time yield}=\frac{Cytotoxic\;capacity\;\left[cells\right]}{V\;\left[mL\right]\times t\;\left[hours\right]}$$ (4)

Equation 4 Calculation of the space-time yield, where the cytotoxic capacity represents the total cytotoxic cells at an E:T ratio of 1:1 at a selected timepoint, V is the total cultivation medium volume used for expansion and t is the process duration.

### Medium analysis

From the spent medium, the concentration of glucose, lactate, glutamine, lactate dehydrogenase (LDH), pyruvate and ammonia was determined by a CEDEX BioHT analyzer (Roche, Basel, Switzerland). The in-line pH was also verified at-line after re-equilibrating the spent medium in a 5% CO_2_ incubator environment for at least 1 h to minimize pH shifts caused by the degassing of CO_2_. Afterwards, the pH was measured immediately after removing the sample from the incubator, using a freshly calibrated pH sensor (Xylem Analytics, San Diego, United States of America). Cell specific uptake and production rates were calculated as described in Equation 5.

$$r=\frac{\left(C_{i}\;*\;V_{i}\right)-\left(C_{i-1}\;*\;V_{i-1}+C_{F,i-1}\;*\;V_{F,i}\right)}{\left(\left(t_{i}-t_{i-1}\right)\;*\;\left(V_{i}-V_{i-1}\right)/2\right)}$$ (5)

Equation 5 Volumetric uptake and production rate r of metabolites and nutrients. $C_{i-1}$ denotes the analyte concentrations in the bioreactor at the current and previous sampling time points, respectively; $V_{i}$ and $V_{i-1}$ are the corresponding reactor volumes; $C_{F,i-1}$ is the analyte concentration in the feed at the previous time point; and $V_{F,i}$ is the feed volume added between sampling points. The denominator accounts for the time interval $\left(t_{i}-t_{i-1}\right)$ and the average reactor volume over that interval.

### Data visualization

Experimental data were exported as comma-separated values (CSV) files and imported into Microsoft Excel (Microsoft Corp., Redmond, WA, United States of America) for analysis and visualization. Graphs were generated using Excel’s built-in charting tools to represent key process parameters and trends across experimental conditions. Data formatting, axis scaling, and labeling were standardized to ensure consistency and clarity across figures. The resulting plots were used for qualitative assessment and comparison of bioprocess performance metrics. Bioreactor visualizations were made using Autodesk Inventor Professional (Version 2024) (Autodesk, Inc. San Francisco, United States of America) and biorender.com (BioRender Science Suite Inc., Toronto, Canada).

### Computational fluid dynamics

The fluid flow field was resolved using the lattice Boltzmann method (LBM), a mesoscopic computational fluid dynamics approach derived from the Boltzmann equation (Succi, 2001; Krüger et al., 2017). All simulations were conducted with the LBM flow solver developed by SimVantage GmbH. The solver utilizes a GPU-accelerated, transient D3Q27 BGK formulation (Bhatnagar et al., 1954), optimized for stirred reactor applications.

For turbulent flow regimes, unresolved sub-grid turbulence was modeled using a Smagorinsky–Lilly large-eddy simulation (LES) approach (Smagorinsky, 1963; Huidan et al., 2005). This approach has been successfully applied and validated for stirred tank and bioreactor applications (Witz et al., 2016).

Aerated simulations additionally resolved the dispersed gas phase using a Lagrangian bubble tracking approach coupled to the LBM-resolved liquid flow field. Bubble motions were modeled following a previously validated framework (Witz et al., 2016).

No-slip boundary conditions were applied at all stationary vessel walls using a bounce-back scheme. Rotating impeller surfaces were modeled using a modified bounce-back formulation for moving boundaries (Ladd, 1994). The free liquid surface was modeled using a VOF-based free-surface lattice Boltzmann method, with interface reconstruction and curvature estimation derived from the volume fraction field according to Bogner et al., 2016 (Bogner et al., 2016).

To characterize the hydrodynamic microenvironment of microorganisms, a Lagrangian passive tracer model was applied. Dispersed particles representing microorganisms were tracked individually in a one-way coupled manner, assuming negligible feedback on the liquid phase. This assumption is valid for microorganisms in the micrometer range at low Stokes numbers. The resulting particle lifelines enable time-resolved exposure analysis from the microbial point of view (Haringa, 2023).

## Results

### NK-92 cell growth and cytotoxicity in pitched-blade and membrane stirrer bioreactors

**FIGURE 2:**
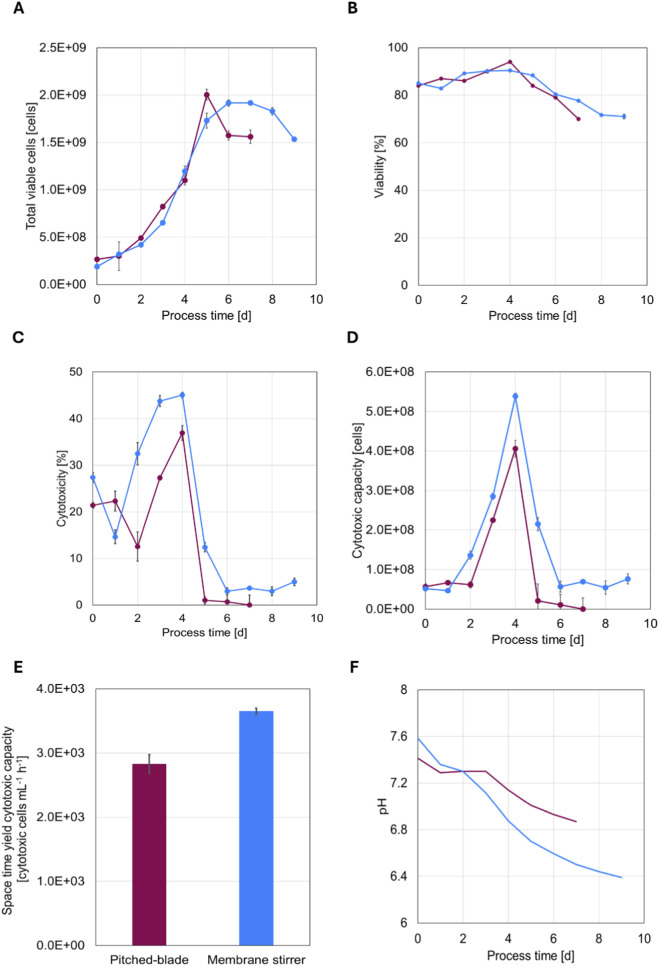
NK-92 total viable cell count **(A)** and viability **(B)** over a 9-day cultivation period in two independent runs using the membrane stirrer (blue), compared to a dual pitched-bladed impeller (purple). NK-92 cytotoxicity **(C)** against K562 target cells (E:T ratio 1:1) over a 9-day cultivation period as well as the cytotoxic capacity **(D)**, space-time yield of the cytotoxic capacity **(E)**, and inline pH **(F)** in both systems. Standard deviation is calculated from techncial replicates (n = 3) of each bioreactor run (n = 1 for each system).

NK-92 cells were cultivated in separate 2 L bioreactors equipped with either conventional dual pitched-blade impellers or the membrane stirrer combination (Figures 1A,B). Because the pitched-blade culture exhibited a longer lag phase (48 h due to prolonged pre-culture), data from both bioreactors were aligned to the beginning of the exponential growth phase for comparison (i.e., the first 2 days of cultivation were excluded from analysis). Total viable cell count and viability over the cultivation period (Figures 2A,B) showed that both systems supported exponential growth after inoculation and achieved comparable peak cell numbers (2.0 × 10^9^ ± 1.7 × 10^7^ cells for the pitched-blade and 1.9 × 10^9^ ± 3.3 × 10^7^ cells for the membrane stirrer). The membrane stirrer culture exhibited a smoother and more gradual transition from the exponential to the stationary phase (after day 6), whereas the pitched-blade culture showed a rapid decline immediately after reaching the exponential-phase peak (on day 5). Viability remained above 80% throughout the growth phase in both systems, followed by a gradual decline into the stationary phase, occurring on day 4 in both systems. Cytotoxicity against K562 target cells at an effector-to-target (E:T) ratio of 1:1 decreased slightly for 1 day at the onset of the exponential growth phase as the cells adapted to the new bioreactor environment, before steadily increasing to a maximum on day 4 in both systems (Figure 2C). Notably, cells cultivated in the membrane stirrer equipped bioreactor achieved markedly higher cytotoxicity values with 36.9% ± 1.6% in the pitched-blade and 45.1% ± 0.6% in the membrane-stirrer setup, respectively. Cytotoxic capacity, defined as the product of the total viable cell count and cytotoxicity, followed a comparable trend, increasing steadily after a short adaptation phase and peaking on day 4 of culture (Figure 2D). The overall cytotoxic capacity was approximately 32% higher in cells expanded in the membrane-stirrer system than in those grown with the pitched-blade impeller (4.06 × 10^8^ ± 7.45 × 10^5^ vs. 5.39 × 10^8^ ± 3.24 × 10^5^, respectively). When normalized to the total culture volume including the feed of fresh medium and taking the process duration into account, the cytotoxic space-time yield was also markedly higher in the membrane stirrer on day 4, confirming increased productivity of functional NK-92 cells (Figure 2E). The pH in both systems was maintained at 7.3 ± 0.05 through controlled feeding of fresh medium, then declined once feeding concluded (on day 3 for the pitched-blade system and day 2 for the membrane stirrer) (Figure 2F).

### NK-92 cell metabolism in pitched-blade and membrane stirrer bioreactors

**FIGURE 3:**
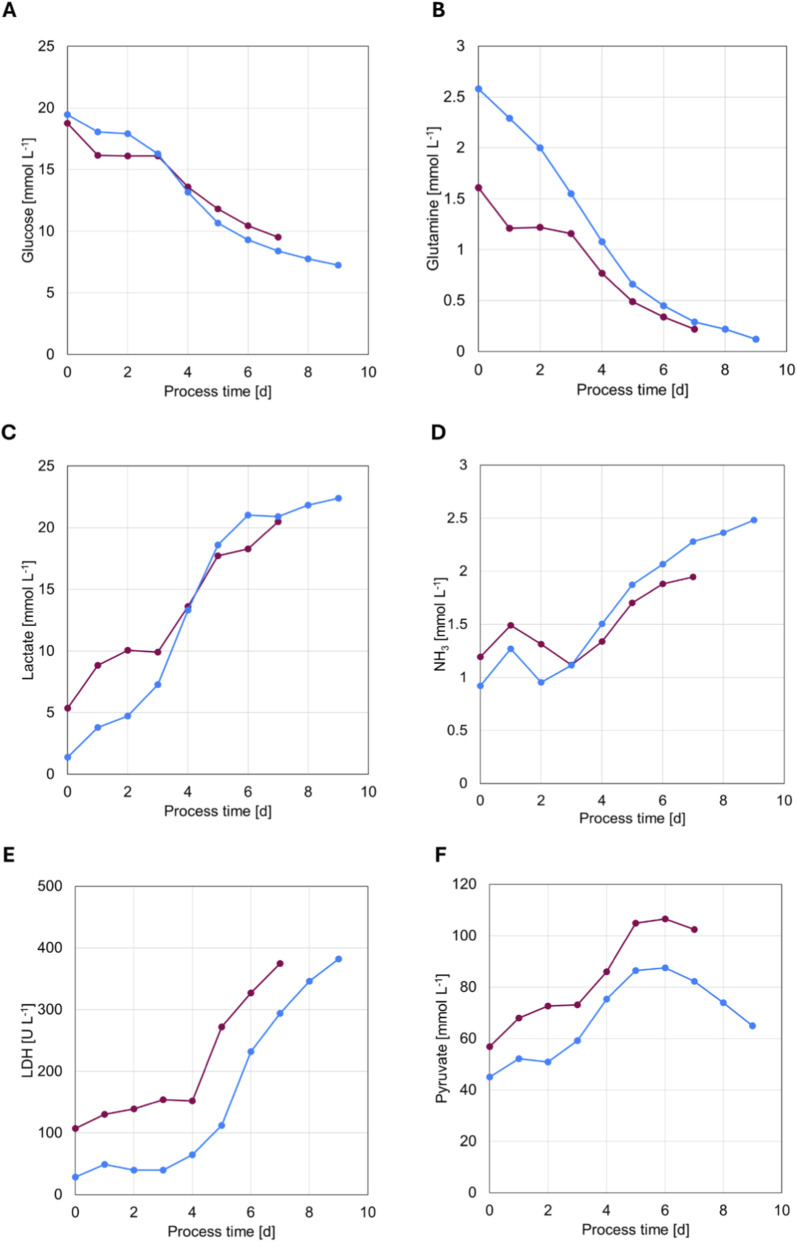
Metabolite concentration kinetics during NK-92 cell expansion over a 9-day cultivation period comparing the membrane stirrer (blue) and a dual pitched-bladed impeller (purple) in 2 L stirred tank bioreactors. **(A)** Glucose, **(B)** Glutamine, **(C)** Lactate, **(D)** ammonia (NH_3_), **(E)** Lactate dehydrogenase (LDH), and **(F)** Pyruvate.

Cells in both bioreactor systems displayed broadly comparable metabolic behavior throughout cultivation. Glucose and glutamine were steadily consumed (Figures 3A,B) at similar consumption rates during exponential growth (Supplementary Figures S2A,B), and did not reach limitation during the growth phase. Lactate and ammonia accumulated to comparable final concentrations (22.4 mM and 20.49 mM Lactate and 2.48 mM and 1.95 mM ammonia in the membrane stirrer and pitched blade system, respectively) (Figures 3C,D) with comparable per-cell production rates (Supplementary Figure SA–D). LDH activity increased steadily in both systems to a final of 382 U/mL and 375 U/mL in the membrane stirrer and pitched blade system, respectively (Figure 3E; Supplementary Figure 2E), while pyruvate levels rose moderately before reaching a plateau on day 5 (86.47 mM and 104.98 mM in the membrane stirrer and pitched blade system, respectively) (Figure 3F). The corresponding specific pyruvate rate declined gradually after around 2 days of cultivation (Supplementary figure 2F). Minor early deviations, particularly in glutamine, lactate, and LDH, resulted from aligning the onset of exponential growth between runs. The pitched-blade culture was shifted by 2 days due to the prolonged pre-culture phase in shake flasks prior to bioreactor inoculation. This extended pre-culture likely led to partial accumulation of metabolic by-products before transfer into the bioreactor, contributing to a prolonged lag phase after inoculation. Overall, metabolic profiles were comparable between the two bioreactor configurations, implying that the observed performance differences arose from factors beyond basic metabolism.

### Shear rates in pitched-blade and membrane stirrer bioreactors

To assess the hydrodynamic environment experienced by NK-92 cells in both bioreactor configurations, computational fluid dynamics (CFD) simulations were performed to quantify liquid velocity fields and shear rate distributions.

**FIGURE 4:**
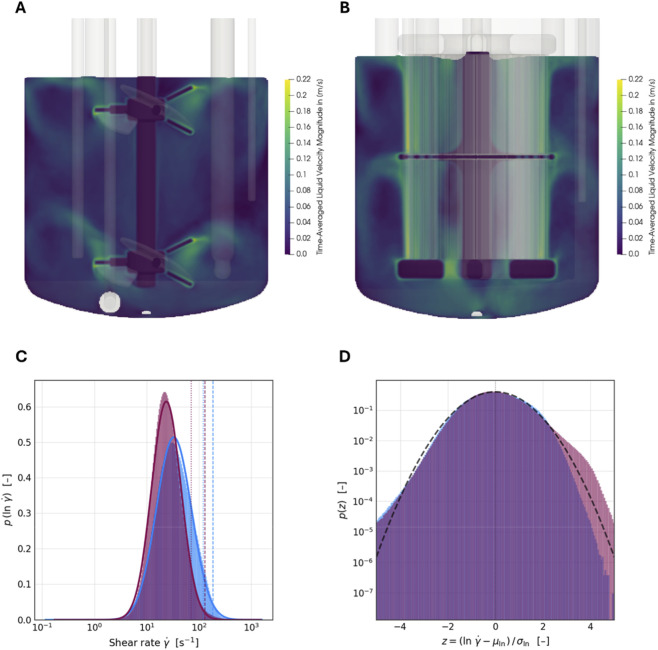
CFD simulations for 2 L bioreactor configurations. Time-averaged liquid velocity magnitude in a stirred-tank bioreactor equipped with a dual pitched-blade impeller **(A)** or membrane-stirrer **(B)** Simulation results were extracted in one second intervals and averaged over a period of 40 s. Probability density function (PDF) of local shear rates calculated from CFD simulations for both configurations, time averaged after reaching the steady state (membrane stirrer in blue, pitched-blade impeller in purple). **(C)** shear-rate distribution in linear space; vertical lines indicat the 95th and 99th percentiles (x95 and x99). **(D)** log-space standardized distributions highlighting distribution shape differences. The membrane stirrer shows near-zero skewness (0.028) and slightly negative kurtosis (−0.23), whereas the pitched-blade system exhibits positive skewness (+0.281) and kurtosis (+0.434), reflecting stronger right-tail behavior and greater shear heterogeneity.

The pitched-blade impeller generated a highly heterogeneous flow field characterized by localized regions of elevated liquid velocity at the impeller tips, while large portions of the vessel exhibited comparatively low flow velocities (Figure 4A). In contrast, the membrane stirrer produced a more homogeneous radial flow pattern, with liquid velocities distributed evenly along the membrane filaments and throughout the bulk volume, despite operating at half the rotational speed of the pitched-blade system (Figure 4B).

Analysis of the shear rate probability density functions revealed distinct differences between both configurations (Figures 4C,D). The membrane stirrer exhibited a slightly higher volume-averaged shear rate and a rightward shift of the distribution (Figure 4C). The pitched-blade system pronounced localized shear gradients near the impeller blades.

These differences are further supported by the log-space standardized distributions (Figure 4D). The pitched-blade system showed bigger skewness than the membrane stirrer system and a positive kurtosis, indicating a heavier right tail and more pronounced extreme shear events. In contrast, the membrane stirrer displayed near-zero skewness and slightly negative kurtosis, reflecting a more symmetric and less heavy-tailed distribution. Together, these statistical descriptors confirm, that the pitched-blade configuration generates a more heterogeneous and intermittent shear environment, whereas the membrane stirrer provides a more spatially uniform shear field.

Importantly, these results indicate that the pitched-blade impeller exposes cells to highly intermittent shear environments, with repeated transitions between low-shear bulk regions and intense localized shear near the impeller tips. In contrast, the membrane stirrer subjects cells to a more continuous and spatially uniform shear environment, reducing the spatial confinement of extreme shear gradients and rapid fluctuations in mechanical stress. The membrane stirrer, by distributing momentum along extended membrane filaments throughout a larger hydrodynamically active volume, spreads energy dissipation more evenly across the reactor. Consequently, a greater fraction of the vessel volume contributes to the overall shear field, shaping both the magnitude and spatial structure of shear exposure. Such differences in shear heterogeneity and exposure dynamics are likely more relevant for shear-sensitive immune cells than volume-averaged shear rates alone and provide a plausible mechanistic explanation for the enhanced cytotoxicity observed in the membrane-stirrer cultures.

## Discussion

This benchmark study comparing fed-batch expansions of NK-92 cells in two bioreactor configurations, a conventional pitched-blade impeller with microsparger and an integrated membrane-based aeration stirrer combination, revealed several important insights into the cultivation of shear-sensitive immune cells at clinically relevant scale.

Both bioreactor designs supported exponential proliferation and achieved comparable peak cell counts (2.0 × 10^9^ ± 1.7 × 10^7^ cells in the pitched-blade system and 1.9 × 10^9^ ± 3.3 × 10^7^ cells in the membrane-stirrer system). These values are exceeding those reported for other currently used GMP-compatible NK-cell expansion platforms such as gas-permeable culture bags (1.36–1.56 × 10^9^ cells in 15–17 days) (Tam et al., 2003), G-Rex® devices (7.5 × 10^8^ cells in 8 days) (Lapteva et al., 2012), and automated systems like the CliniMACS Prodigy® (1.3 ± 0.9 × 10^9^ cells in 14 days) (Granzin et al., 2015). Our results further demonstrate the strong potential of stirred-tank bioreactors for NK-cell manufacturing, as the overall production time was markedly shorter compared to all other platforms resulting in more cytotoxic NK cells. Across both tested systems, metabolic profiles remained stable and comparable, confirming that sufficient gas transfer was maintained at 2 L scale. Prior to inoculation the volumetric mass-transfer coefficient (kLa) was determined *via* the off-gas method and maintained at 0.3 h^-1^ in both systems during cultivation, using identical aeration rates (0.02 vvm) but lower agitation in the membrane-stirrer (40 rpm vs. 80 rpm in the pitched-blade setup) (Bauer et al., 2024). Despite similar growth trajectories, the membrane-stirrer culture exhibited a more gradual transition from exponential to stationary phase, whereas viable cell density in the pitched-blade system declined sharply immediately after reaching its peak. This difference coincided with a pronounced improvement in cell functionality in the membrane-stirrer bioreactor: both cytotoxicity and cytotoxic capacity were markedly higher, with the latter reaching approximately 32% above the pitched-blade culture.

Given the reduced agitation speed and bubble-free aeration in the membrane-stirrer system, the enhanced NK-92 cytotoxicity is likely linked to differences in shear distribution and exposure dynamics rather than to changes in average shear magnitude. Importantly, no antifoam or shear-protective additives (e.g., poloxamers) were added in either the pitched-blade/microsparger system or the membrane-stirrer configuration. Both bioreactor cultures were conducted entirely without such supplements to deliberately isolate the effects of agitation and aeration strategy on NK-92 cell performance. Consequently, the observed differences in cytotoxicity cannot be attributed to the presence or absence of shear- or foam-reducing agents but rather reflect the intrinsic hydrodynamic characteristics of the respective systems. Importantly, the quantified shear rates in both stirred-tank configurations remained comparably low and were approximately an order of magnitude lower than those reported for commonly used shake-flask systems (Giese et al., 2014). Prior studies have demonstrated that localized and transient shear stresses, particularly those associated with gas bubbles and impeller regions, can negatively affect immune cell performance (Ma et al., 2004; Hu et al., 2008; Flynn et al., 2024; Bongartz et al., 2023; Walls et al., 2017). While the overall low shear environment in both systems supported strong NK-92 cell growth, the more homogeneous shear-rate and fluid-velocity distribution in the membrane-stirrer configuration appears to be beneficial for preserving and enhancing cytotoxic function. The enhanced cytotoxic capacity translated directly into a higher cytotoxic space-time yield, highlighting a key benefit for future manufacturing of “off-the-shelf” allogeneic NK-cell therapies in this bioreactor system. Although direct evidence linking shear distribution to NK-cell cytotoxicity remains limited, emerging data suggest plausible biological mechanisms. A recent study using vessel-mimicking dynamic conditions reported enhanced NK-cell cytotoxicity under homogeneous shear environments (Tran et al., 2024). Furthermore, fluid shear stress has been shown to activate the NKG2D receptor, promoting NK-cell activation and degranulation (Hu et al., 2023). Together, these findings indicate that controlled mechanical stimulation can modulate NK-cell functional responses. It is conceivable that a homogeneous shear environment provides beneficial mechanical cues that sustain activation signaling while avoiding localized, transient high-shear events that may impair receptor integrity or immune synapse formation (Santoni et al., 2021). However, as this study focused primarily on technical feasibility and scalability, mechanistic investigations of shear-induced signaling pathways were beyond its scope. Future studies should therefore examine receptor expression, intracellular signaling, and degranulation dynamics to clarify the causal link between shear distribution and enhanced cytotoxic performance.

For this study, an E:T ratio of 1:1 was selected to assess cytotoxicity, as previous reports demonstrated that this ratio provides robust and sensitive detection of NK-cell–mediated killing after 4 h of co-incubation, with improved assay sensitivity compared to longer incubation periods (e.g., 24 h) (Langhans et al., 2005). This experimental setup has also been broadly applied in NK-cell research over the past decades to quantify cytotoxic function (Bazhenov et al., 2020; Xu et al., 2025; Saito et al., 2013; Eskandarian et al., 2026). K562 cells are frequently used in NK-cell assays due to their consistent susceptibility to NK-cell–mediated lysis and their stable growth characteristics, ensuring that reductions in viability predominantly reflect effector-cell activity rather than variability in target cell culture conditions. While this assay provides reliable primary insight into NK-92 functional potency, further evaluation against additional tumor cell lines (e.g., Raji, RS4; 11, or Jurkat) and, ultimately, *in vivo* models would be valuable to more comprehensively assess cytotoxic performance, particularly in a clinically relevant context.

Importantly, the observed functional improvements translated into measurable process advantages at production scale. From a manufacturing perspective, achieving a higher number of functionally potent cells (i.e., increased cytotoxic capacity) within the same bioreactor footprint and process time is critical for reducing costs and meeting the increasing demand for NK-cell–based immunotherapies. Here, the membrane-stirrer bioreactor provided an advantage without compromising scalability, or process simplicity. It should be noted that a limitation of this study is that only one independent bioreactor run was performed per configuration. However, in our previous studies with the NK-92 cell line, independent experimental repetitions under static and manually handled culture conditions demonstrated negligible standard deviations in growth characteristics and cytotoxicity (von Werz et al., 2026). Moreover, the tight control of critical process parameters such as pH, dissolved oxygen, temperature, and agitation in closed bioreactor systems generally results in enhanced reproducibility compared to conventional culture platforms. On this basis, a single representative run per condition was considered scientifically justifiable for this comparative benchmark study. Nevertheless, to further strengthen generalizability and robustness, additional independent replicates would be desirable in future investigations.

In summary, while both bioreactor configurations are suitable for clinically relevant NK-92 expansion, the membrane-stirrer design offers a more favorable cultivation environment characterized by homogeneous shear distribution and elimination of bubble-dependent aeration and foam formation. This resulted in substantially enhanced cytotoxic function and process productivity compared to static and manual expansion (von Werz et al., 2026), exceeding the potency critical quality attribute ranges of current clinical trials (von Werz et al., 2025). Importantly, both systems produced approximately 2 × 10^9^ viable NK-92 cells without impaired growth or functionality, underscoring the feasibility of further intensification and scale-up toward larger bioreactor volumes needed for clinical manufacturing (Sakamoto et al., 2015; Marin et al., 2024). Looking ahead, integrating perfusion strategies, automated in-line monitoring, and advanced process-control frameworks may further enhance process robustness and functional performance, supporting large-scale, cost-efficient production of therapeutic NK cells.

## Data Availability

The original contributions presented in the study are included in the article/Supplementary Material, further inquiries can be directed to the corresponding authors.
