# Supplementary material for: Homogeneous shear distribution improves NK-92 cell cytotoxicity in a clinically relevant 2 L membrane-stirred bioreactor
Source: Front Bioeng Biotechnol. 2026 Mar 23;14:1804945. doi: 10.3389/fbioe.2026.1804945 (PMC13050783; doi:10.3389/fbioe.2026.1804945)
Supplement: Supplementary file 1 [file DataSheet1.pdf]

Supplementary Figures

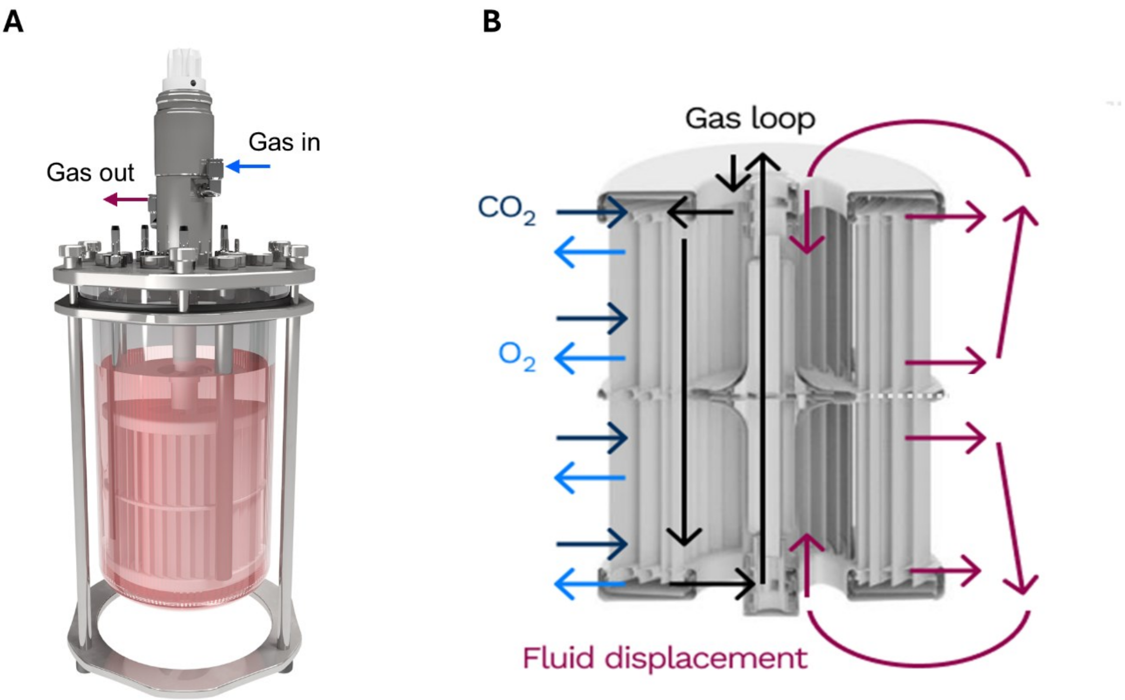

Supplementary figure 1: Schematic representation of the membrane stirrer in a 2 L glass bioreactor vessel (A). Via the patented fluid-wave coupling mechanism, process gases are introduced on one side (blue arrow) and exit through the opposite side (purple arrow). The membrane stirrer side cross section (B) illustrates the open-loop gas flow (blue and black arrows) as well as the fluid displacement (purple arrows). The membrane blades consist of multiple fixed hollow fibers enlarging the total surface for optimal gas transfer [WO2021152128 and WO2024240589A1]. The rendering was built with Inventor® 3D-CAD-Software.

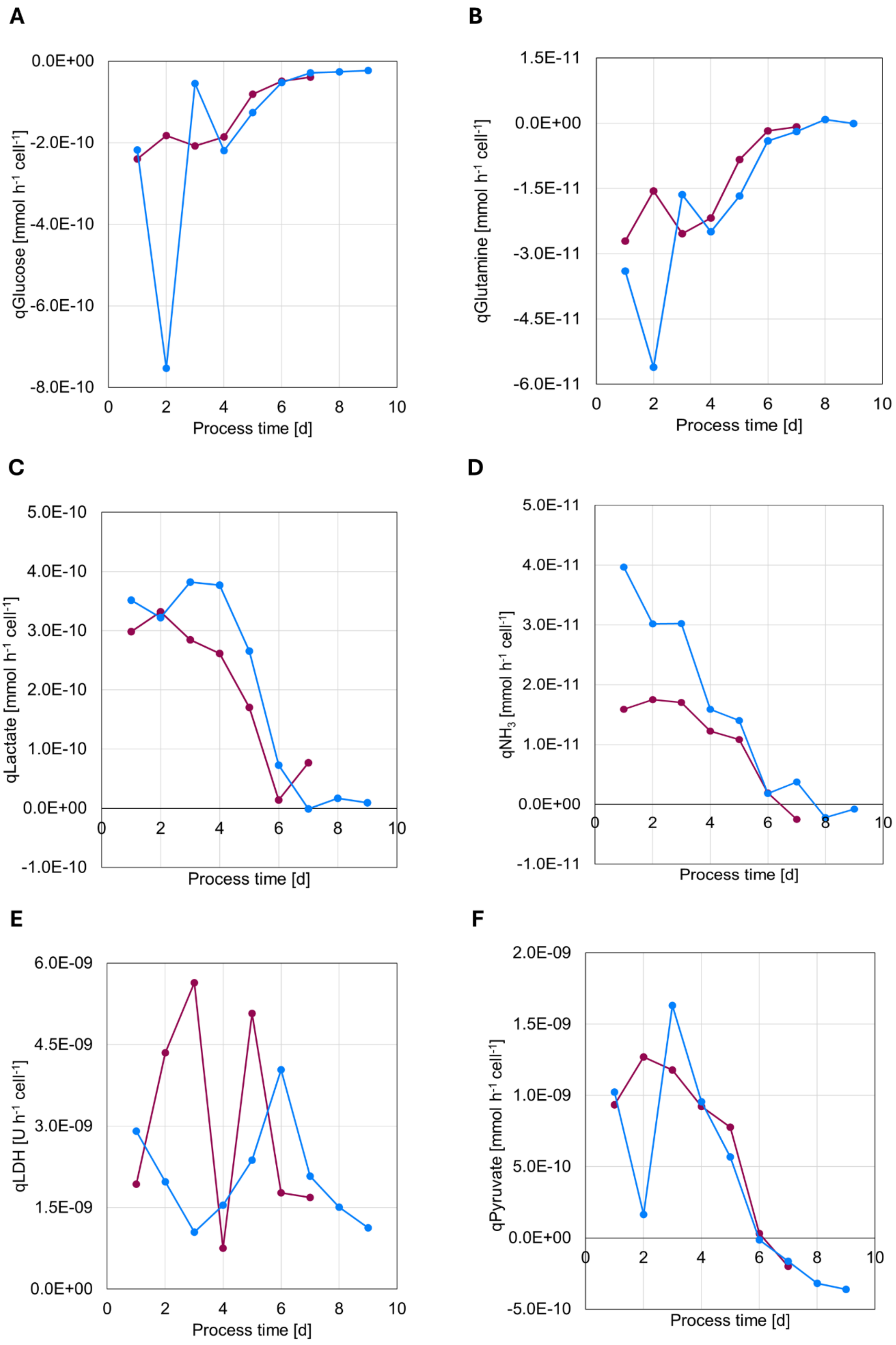
A
0.0E+00
-2.0E-10
-4.0E-10
-6.0E-10
-8.0E-10
qGlucose [mmol h-1 cell-1]
0 2 4 6 8 10
Process time [d]
B
1.5E-11
0.0E+00
-1.5E-11
-3.0E-11
-4.5E-11
-6.0E-11
qGlutamine [mmol h-1 cell-1]
0 2 4 6 8 10
Process time [d]
C
5.0E-10
4.0E-10
3.0E-10
2.0E-10
1.0E-10
0.0E+00
-1.0E-10
qLactate [mmol h-1 cell-1]
0 2 4 6 8 10
Process time [d]
D
5.0E-11
4.0E-11
3.0E-11
2.0E-11
1.0E-11
0.0E+00
-1.0E-11
qNH3 [mmol h-1 cell-1]
0 2 4 6 8 10
Process time [d]
E
6.0E-09
4.5E-09
3.0E-09
1.5E-09
0.0E+00
qLDH [U h-1 cell-1]
0 2 4 6 8 10
Process time [d]
F
2.0E-09
1.5E-09
1.0E-09
5.0E-10
0.0E+00
-5.0E-10
qPyruvate [mmol h-1 cell-1]
0 2 4 6 8 10
Process time [d]

Supplementary figure 2: Metabolite per-cell production and uptake rates (qMetabolite) kinetics during NK-92 cell expansion over a 9-day cultivation period in two independent 2 L stirred-tank bioreactors equipped with a membrane stirrer (blue) or a dual pitched-blade impeller and microsparger (purple). (A) qGlucose, (B) qGlutamine, (C) qLactate, (D) qAmmonia (NH_3), (E) qLactate dehydrogenase (LDH), and (F) qPyruvate.

Supplementary table 1 Process parameters used in the bioreactors equipped with a pitched-blade impeller or membrane stirrer.

Process parameter	Pitched-blade impeller	Membrane stirrer
**Impeller type**	Two *30° 3-blade pitch-blade impellers*	Membrane stirrer
**Aeration method**	Microsparger	Membrane aeration
**Temperature**	37°C	37°C
**Gasmix flow rate**	12mL/min	12mL/min
**Dissolved oxygen**	60%	60%
**pH**	7.35 ± 0.05	7.35 ± 0.05
**Agitation speed**	80 rpm	40 rpm
**Antifoam**	Not used	Not used
**kLa**	*0.3 h^{-1}*	*0.3 h^{-1}*
